# Supplementary material for: Predicting visual function by interpreting a neuronal wiring diagram
Source: Nature. 2024 Oct 2;634(8032):113–23. doi: 10.1038/s41586-024-07953-5 (PMC11446822; doi:10.1038/s41586-024-07953-5)
Supplement: Supplementary file 6 — CRF and ERF predictions for individual TmY4 and TmY9 cells. Analogous to Supplementary Data 3, but for TmY target types. Shown are the top four monosynaptic pathways, the strongest pathway passing through each of the top ten intermediary types (ranking from Extended Data Fig. 7), and the trisynaptic pathway Tm1–TmY–Dm3–TmY (see the section entitled Prediction of spatial normalization). [file 41586_2024_7953_MOESM6_ESM.zip › DataS4/TmY9qΓèÑ/720575940633195079.pdf]

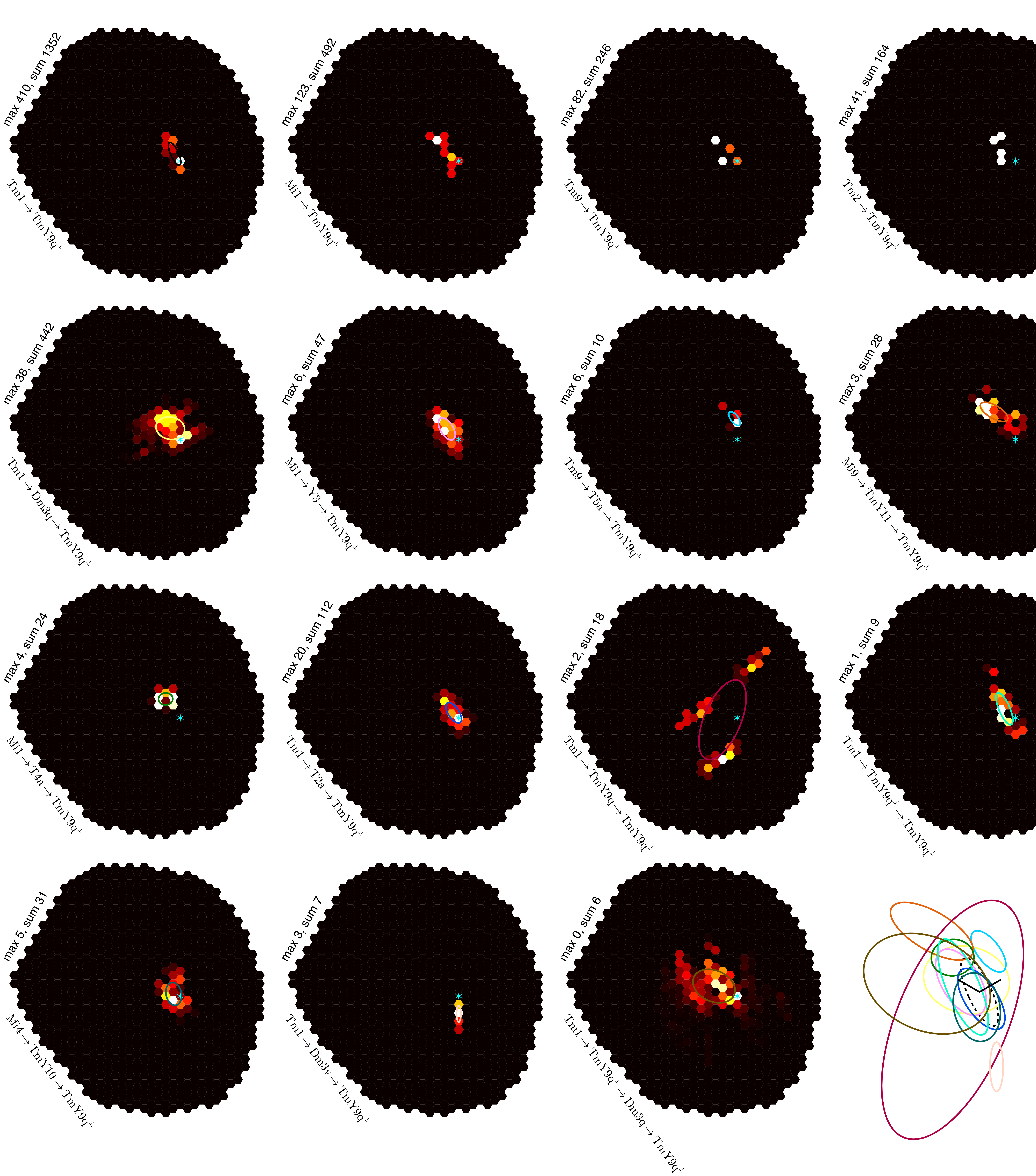
max 410, sum 1352
Tm1 → TmY9q⊥
max 123, sum 492
Mi1 → TmY9q⊥
max 82, sum 246
Tm9 → TmY9q⊥
max 41, sum 164
Tm2 → TmY9q⊥
max 38, sum 442
Tm1 → Dm3q → TmY9q⊥
max 6, sum 47
Mi1 → Y3 → TmY9q⊥
max 6, sum 10
Tm9 → T5a → TmY9q⊥
max 3, sum 28
Mi9 → TmY11 → TmY9q⊥
max 4, sum 24
Mi1 → T4a → TmY9q⊥
max 20, sum 112
Tm1 → T2a → TmY9q⊥
max 2, sum 18
Tm1 → TmY9q → TmY9q⊥
max 1, sum 9
Tm1 → TmY9q⊥ → TmY9q⊥
max 5, sum 31
Mi4 → TmY10 → TmY9q⊥
max 3, sum 7
Tm1 → Dm3v → TmY9q⊥
max 0, sum 6
Tm1 → TmY9q⊥ → Dm3q → TmY9q⊥
